# Barriers to the implementation of public-private partnerships in the healthcare sector in the Kingdom of Saudi Arabia

**DOI:** 10.1371/journal.pone.0233802

**Published:** 2020-06-18

**Authors:** Mohammed Khaled Al-Hanawi, Sarh Almubark, Ameerah M. N. Qattan, Agnieszka Cenkier, Ewa Agnieszka Kosycarz

**Affiliations:** 1 Department of Health Services and Hospital Administration, Faculty of Economics and Administration, King Abdulaziz University, Jeddah, Saudi Arabia; 2 Collegium of Business Administration, SGH Warsaw School of Economics, Warsaw, Poland; 3 Collegium of Socio-Economics, SGH Warsaw School of Economics, Warsaw, Poland; University of Lincoln, UNITED KINGDOM

## Abstract

**Background:**

Saudi Arabia is considering increasing the role of the private sector’s participation in financing and delivering healthcare services through the adoption of Public-Private Partnerships (PPPs). However, the adoption and successful implementation of PPPs in the Saudi healthcare sector requires careful attention to overcome potential obstacles.

**Objectives:**

This study investigates and identifies potential barriers to the successful implementation of PPPs in the Saudi healthcare sector.

**Methods:**

A pre-tested interviewer-administered questionnaire was used to collect data from 72 respondents over a two-month period. Respondents were asked to rate the degree of influence of potential key barriers using a five-point Likert scale. The collected data was analysed using descriptive and inferential statistics.

**Results:**

The evidence showed that the top three barriers, as rated by the respondents, were legal barriers, including delays in receiving approval and permits and law and regulation changes, environmental barriers, including lack of transparency and accountability and technological barriers, including a shortage of professionals qualified to handle PPP projects.

**Conclusions:**

The barriers identified suggested that the government should ensure that PPPs are implemented in a timely manner to ensure that private sector involvement yields the intended benefits. Furthermore, a stable legal and regulatory framework must be established that is properly and easily enforced to avoid confusing stakeholders with too many changes. It is also important to ensure that transparency and accountability measures are strengthened.

## Introduction

One of the main duties of governments is to provide essential public services. The provision of such services, including education, transport infrastructure and healthcare, requires adequate resources, including financial resources. Governments provide these services by building infrastructure and financing projects delivering other services to the citizenry. When providing these services, governments face many constraints, including technical challenges (because governments might be unable to provide all services effectively and efficiently) and financial challenges (because they do not have unlimited budgets) [1,2]. It is, therefore, natural for governments across the globe to consider new initiatives and one increasingly common method of delivering public services is to form public-private partnerships (PPPs) [3–5].

PPPs have received much attention in the context of the development and financing of public infrastructure facilities and services in the last decade due to their inherent advantages. This approach has been adopted by many countries for several reasons, including the need to address fiscal deficits, budgetary pressure and demand-supply gaps [6]. PPPs are also perceived as a tool that can unite the private and public sectors’ strengths to improve efficiency, quality and innovation [7]. PPPs are commonly used to take advantage of innovations found in the private sector, lower the risks associated with the development of public sector assets and enhance the success of the development and financing of public infrastructure facilities and services for the public [8]. It is argued that PPPs have increased efficiency, the availability of resources and the sustainability of public services in several sectors, including telecommunications, water, energy, transport and health [9].

PPP adoption in the healthcare sector has increased due to the many benefits that are associated with them, both in healthcare development and management [10]. The benefits identified included overcoming the capital shortage in public sectors, reducing the risks of investment in health infrastructure, improving efficiency in service delivery through combining efforts, making use of innovations in the private sector and aligning healthcare stakeholders in meeting healthcare needs [8].

The Kingdom of Saudi Arabia (KSA) is a high-income, developing country [11]. Its economy is extremely dependent on oil revenue, which accounts for over 90% of its exports and approximately 75% of government revenue [12]. The country’s oil wealth has allowed the government to finance public services, including the healthcare sector. However, oil price fluctuations affect government revenue and, consequently, all sectors of the Saudi economy [13].

In KSA, healthcare services are provided through the Ministry of Health (MOH), other government agencies and private healthcare providers. The KSA government is responsible for operating, financing and managing the public healthcare sector, which delivers 80% of all healthcare services, free of charge at the point of use, to Saudi citizens [14]. The private sector provides the remaining healthcare services based on a fee for service, paid for by the patient personally or through private health insurance plans [15].

Despite the substantial resources that the government is currently able to allocate to it, the healthcare system, like most publicly funded healthcare systems is increasingly under strain, caused by rapid increases in expenditure and demand while resources remain finite. Hence, as has been recognised by both academics and international health organizations, relying solely on oil revenues to finance public healthcare services is unsustainable in the medium to long term [16–18].

Recently, the Saudi Government initiated a major economic reform and adopted “Vision 2030” as a roadmap for economic development. Vision 2030 is a strategic plan aimed at reducing Saudi Arabia’s reliance on oil revenue and its priorities have been identified in all economic sectors. It has been implemented with the aim of identifying the general direction, policies, goals and objectives of KSA [19]. One of the initiatives and reforms in the Saudi healthcare sector is the increase in the role of private sector participation in delivering and financing healthcare services through the utilisation of PPPs.

The adoption of PPPs in the Saudi healthcare sector, therefore, requires caution in identifying and attempting to overcome obstacles to their successful implementation in the long term. Hence, it is worth investigating and understanding how to successfully implement PPP in healthcare. This study aims to identify and classify the perceived obstacles and barriers to the successful implementation of PPPs, so that policymakers can take them into consideration as KSA implements the Vision 2030 program. This will allow the government and other stakeholders to recognize the barriers and develop plans and solutions that will prevent them from consistently besetting progress in the healthcare sector. This research adds to the existing body of knowledge on barriers to the implementation of PPPs in the healthcare sector.

### The concept of PPPs in healthcare

Cooperation between the public and private sectors in healthcare infrastructure construction and maintenance and healthcare services delivery has been longstanding and takes various forms, from outsourcing to almost complete privatisation of services [20]. PPPs in healthcare were launched in the early 1990s and they redefined the model of cooperation between the public and private sectors [7]. Governments introduced PPPs to improve value for money and to attract private capital to produce public goods and services. The popularity of PPPs is also a result of the emergence of alternative instruments in public funds management focused around New Public Management [21]. As the European Commission's report indicates, there have been changes in the perception of state institutions from that of a service provider to a regulator, controller and organiser of the system for the provision of public services and goods [22].

There is no single precise definition of PPPs [23–25]. According to the World Bank Group’s broad definition, a PPP is “a long-term contract between a private party and a government entity to provide a public asset or service, in which the private party bears significant risk and management responsibility, and remuneration is linked to performance” [26]. In practice, there are many different models of such partnerships, including institutional cooperation for joint production and risk sharing, long-term infrastructure contracts that emphasise tight specific of outputs, public policy networks, civil society and community development and urban renewal and downtown economic development [24]. Additionally, there are several PPP models in the healthcare area, including build-operate-transfer (BOT), build-own-operate (BOO), build-own-operate-transfer (BOOT) and design-build-finance-maintain (DBFM) [9].

PPPs have been used to encourage and facilitate innovations in many healthcare fields. PPPs in public health research [27] have included finding vaccines against, and drugs to treat, communicable diseases [28,29], personalised medicine development [30] and management and infrastructure growth [31,32]. The biggest controversies and expectations concern building and operating healthcare infrastructure, such as hospitals. In developed countries, PPP models based on designing, building and maintaining healthcare infrastructure prevail and the service provision is often left to the public partner [33]. However, in some solutions, healthcare services are provided in facilities owned and maintained by a public entity [7].

### Advantages and disadvantages of PPPs in healthcare

There are several advantages of PPPs in healthcare, including increasing efficiency, allocating risk, reducing the cost of delivery quality services, increasing accessibility, delivering services on time, adopting new technology and management, solving public sector capital shortages and reducing fiscal and public pressures [20,34].

For the sake of efficiency, it is estimated that the optimal solution is where a private entity is responsible for a healthcare facility’s design, construction and continuing maintenance [20]. It is expected that the private entity will put more effort into initial design and construction in order to optimise life-cycle costs and reduce operating and maintenance costs. Higher efficiency of service delivery in PPPs is associated with the private sector’s innovative strength and advanced management skills. The private sector also has a better incentive system [35].

A great advantage of PPPs is proper risk-sharing between the partners. There are many types of risks in healthcare contracts, including demand, demographic change, health changes, epidemiological trends, long-term healthcare demands, infrastructure construction risks, operational risks, and financial risks [35]. Proper risk-sharing is a key issue. If properly implemented, the PPP model can result in better investment decisions and can reduce the total cost of delivering services of a certain quality [36–38]. Barlow and Köberle-Gaiser [39], based on an analysis of PPPs in hospitals, suggested inadequate risk allocation as a reason for abandoning innovation. Additionally, many countries’ experiences have shown that PPPs in healthcare have helped to reduce fraud by increasing accountability, transparency and control [40]. Such an effect could be expected in countries where the risk of corruption is very high [41].

In spite of the advantages of PPPs, their implementation still suffers from several disadvantages and impediments, including higher costs of providing goods and services, difficulty in specifying services (due, for example, to innovations in medical equipment) resulting in difficulty in quantifying the cost of PPP projects, political risks associated with long-term cooperation between public and private sectors, volatile and often unpredictable external conditions (e.g., epidemiological risks), difficult and often inappropriate infrastructure maintenance and service delivery pricing, lengthy procurement processes, lack of appropriate skills, unattractive financial markets and incomplete risk transfer and higher end user charges [42–44].

PPPs in healthcare projects also experience many impediments that characterise other PPP investments, including lack of suitable skills and experience, lengthy bidding and negotiation processes, lack of well-established legal framework, legal risks, unfavourable economic and commercial conditions, inefficient public procurement frameworks, lack of mature financial engineering techniques, public opposition, delays because of political debate and lack of competition [9,45].

Both public and private partners in PPP arrangements should work to overcome the barriers and try to ensure that such issues do not hinder the proposed projects’ progress [46]. One of the most important issues for successful long-term PPPs is to determine and ensure robust contractual and relational governance mechanisms [47]. This is indirectly related to the type of health PPP model. For example, private finance initiative contracts are the least flexible in the longer term. There are no incentives for the private partner to make any required changes, which could have a negative impact on the public-private relationship [48].

### Healthcare sector PPPs in KSA and the need to study barriers to their success

The Saudi MOH is committed to providing healthcare services that meet the needs of the citizenry and has made a commitment to achieve this objective, in part, by empowering the private sector to participate in the healthcare sector’s transformation through partnerships in the financing of capital and realisation of operational projects [49]. It is anticipated that this will improve the healthcare sector’s efficiency, rationalise financial expenditures and reduce the burden on the public budget. Accordingly, the MOH has introduced two pilot projects, one in radiology and the other in service provision [49].

These two projects are currently at the pilot stage and limited information about them is available. The radiology project is extremely ambitious and is planned for a 10 year period. It will cover demand for radiology services from seven hospitals, as well as nuclear medicine services, serving 1 million beneficiaries. The private partner will get, on a “right of use” basis, the existing infrastructure and qualified staff. The private partner will be responsible for replacing equipment over the project term, for investing in IT infrastructure and networks to set up a tele-radiology solution and any required refurbishment. The payment mechanism will be based on ongoing payments by the MOH and will be on a per-scan basis [50].

Because PPPs have only recently been introduced and the MOH has committed to utilising this model until 2030, KSA provides a unique case in which barriers to the utilisation of PPPs can be studied as they arise, allowing the policies to be reviewed and feedback to be provided during the project’s implementation. Such data will be extremely helpful when the MOH expands the implementation of PPPs in the healthcare sector in the future.

## Materials and methods

We conducted a descriptive study to identify and map barriers to the implementation of PPPs in the Saudi healthcare sector. A pre-tested interviewer-administered questionnaire was used to collect data from 72 stakeholders involved in PPPs in the Saudi healthcare sector over a two-month period (June-July 2019). The target respondents included various stakeholders from both the public and private sectors, i.e. those knowledgeable about the implementation of PPPs in this context. They included government leaders, public sector officials, private investors, consultants, lenders and representatives from contractors involved in PPP projects. Interviewer-administered questionnaire was used in order to accurately record responses, to make the most efficient use of time, and to avoid any missing data. The sample was purposively selected to gather the insights of a varied group of people. The study participants’ main characteristics are presented in Table 1.

**Table 1:** Study participants’ main characteristics.

Item	Category	Frequency (n)	Percentage (%)
Role in Healthcare PPP projects	Government leaders	10	14
	Public sector officials	40	56
	Private Investors	4	6
	Lenders/Financial	6	8
	Consultants	6	8
	Contractors	6	8
Highest academic qualification	High School or below	2	3
	University degree	30	42
	Master degree	32	44
	PhD	8	11
Years of industry experience	0–5 years	28	39
	6–10 years	24	33
	11–15 years	12	17
	≥ 16 years	8	11
Number of PPP projects involved in	1–2	44	61
	3–4	24	33
	5–6	2	3
	≥ 7	2	3

The questionnaires were administered in the Arabic language by the author (SA). The interviewer read, managed and completed the questionnaire based on the answers provided by the participants to record responses accurately and make the most efficient use of time. The interviews lasted between 15–30 minutes, with an average length of 21 minutes. The interviewer-administered questionnaire guide was adapted from the validated questionnaires developed by Babatunde et al. [9] and Li [51].

The questionnaire included two main sections. The first section asked the respondents for general information related to their demographic characteristics and PPP project knowledge and experience. The second section was designed to investigate barriers to the successful implementation of PPPs in the Saudi healthcare sector. Forty-six pre-identified barriers were divided into six categories using the SLEEPT approach (Social, Legal, Economic, Environmental, Political, Technological [9]. The SLEEPT approach is a verified tool providing in-depth analysis of business environments, enabling business and political leaders to build a more efficient and effective system of delivering public goods and services, such as healthcare provisions. Respondents were asked to rate the degree of influence of potential key barriers in each category using a five-point Likert scale (1 = very low; 5 = very high).

The interviews took place in the participants’ offices. They were informed of the study’s aim and objectives, that they had the right to withdraw at any time without giving a reason or explanation and that all data, information and opinions would be confidential and anonymised. Additional informed consent was obtained from all individuals whose identifying information was included in this article.

Descriptive and inferential analyses were used to evaluate data regarding the various barriers and their perceived degree of importance. The measurements of descriptive statistics used in this study included percentages, averages and mean scores, which were used to interpret the distribution of the samples. Mean score ranking was used to evaluate each barrier’s importance, as judged by the respondents. The Kruskal-Wallis test was employed to draw inferences from the data as it is a suitable statistical tool when comparing three or more groups [52]. Here, the Kruskal-Wallis test was conducted to determine whether there were significant differences in the opinions of various stakeholders involved in healthcare-related PPP projects regarding the barriers to the successful implementation of PPPs. All analyses were conducted using Statistical Package for Social Science (SPSS) software.

A reliability test was conducted to test the data-collection questionnaire’s internal consistency. There are several ways to measure the study tool’s reliability; this study used the Cronbach's alpha and split-half methods, mainly the Spearman-Brown and Guttman split-half coefficients [53]. A Cronbach’s alpha value of 0.7 or higher is considered an indicator of adequate reliability [54], as is a split-half correlation coefficient value of 0.8 or higher [55]. The reliability coefficient value of Cronbach’s Alpha was 0.841, the Spearman-Brown’s split-half coefficient was 0.882 and the Guttman’s split-half coefficient was 0.881. Based on these values, the study tool appeared to possess internal reliability.

### Ethical consideration

All procedures performed in studies involving human participants were conducted in accordance with the ethical standards of the institutional and/or national research committee and with the 1964 Helsinki declaration and its later amendments or comparable ethical standards. This study has been reviewed and approved by the King Abdulaziz University Research Ethics Committee. The study was designed and conducted in accordance with the ethical principles established by King Abdulaziz University. In addition to King Abdulaziz University’s ethical approval, the study has also received ethical approval from the MOH in KSA (Central IRB log no. 2019-0058M). Written consent to participate was secured from all respondents who participated in the study.

## Results

The ranking of the barriers to the implementation of PPPs are presented in Table 2, which shows the categorised barriers’ mean rank values using the SLEEPT approach. The mean rank values of the respondents’ categories are also included.

**Table 2:** Mean rank values of barriers by sample groups and their rankings.

		Barriers					
		Social	Legal	Economic	Environmental	Political	Technological
All Categories	Mean	3.23	3.85	3.36	3.55	2.77	3.49
	Rank	5	1	4	2	6	3
Government leaders	Mean	3.42	4.26	4.12	3.96	3.29	3.70
	Rank	5	1	2	3	6	4
Public sector officials	Mean	3.27	3.92	3.31	3.45	2.92	3.55
	Rank	5	1	4	3	6	2
Private Investors	Mean	2.56	3.96	3.56	3.10	1.71	3.45
	Rank	5	1	2	4	6	3
Lenders/Financial	Mean	3.52	3.90	3.17	3.73	3.09	3.77
	Rank	4	1	5	3	6	2
Consultants	Mean	3.44	3.76	3.50	3.73	2.29	3.37
	Rank	4	1	3	2	6	5
Contractors	Mean	3.07	2.67	2.33	3.47	1.71	2.67
	Rank	2	3	5	1	6	3

For each category of barriers, several statements were used (detailed barriers) to assess the barrier’s importance. Details of the statements and their ranking list (based on their mean values) are presented in Table 3.

**Table 3:** Detailed results of the rankings of the barriers to implementing PPPs.

Barriers		Mean	Rank
Social	Potential conflicts of interests among stakeholders	3.81	1
	Inadequate consultation by stakeholders to facilitate greater acceptance of PPPs	3.60	2
	Fear over the implications of decisions	3.57	3
	Lack of trust between the public and private sectors	3.46	4
	Cultural impediments, including the public perception of PPPs	3.20	5
	Inability of the public sector to manage consultants	3.08	6
	Lack of confidence and trust in PPPs	2.94	7
	Societal discontent with the private sector	2.79	8
	Public opposition	2.76	9
Legal	Delays in approval and permits	4.06	1
	Law and regulation changes	3.97	2
	Weak/poor regulatory frameworks and enforcement	3.92	3
	Problems with administrative procedures and guidelines	3.92	4
	Weak/poor enabling policies	3.78	5
	Unavailability of model concession agreements	3.69	6
	Weak institutional capacity and PPP strategies	3.57	7
Economic	Problems with delays in receiving payments	3.64	1
	Inability of local institutions to provide long-term financing	3.53	2
	Financial cost	3.46	3
	Inappropriate risk allocation and sharing	3.44	4
	Perceived rise in tariffs	3.36	5
	Difficulties in securing credit from banks	3.21	6
	Difficulties in obtaining long-term financing	3.20	7
	Lack of public-sector project development funds to promote PPPs	2.94	8
Environmental	Lack of transparency and accountability	3.72	1
	Lengthy delays in negotiations	3.72	2
	Environment that does not facilitate the implementation of PPPs	3.50	3
	Construction delays	3.49	4
	Land-acquisition problems	3.29	5
Political	Inadequate experience with PPPs	3.29	1
	Poor understanding of PPPs by decision-makers	3.22	2
	Distortions of guarantees/incentives by governments	2.97	3
	Lengthy delays because of political debates	2.69	4
	Inability of the government to manage PPP projects	2.50	5
	Strong political interference	2.32	6
	Lack of strong political commitment to PPPs	2.31	7
Technological	Shortage of professionals qualified to handle PPP projects	3.78	1
	PPP process that is not clearly defined/lack of clarity	3.69	2
	Inability of the private sector to fully meet the challenge of investing in many PPP projects	3.60	3
	Inconsistent risk assessment and management	3.59	4
	Inadequate distribution of responsibilities	3.53	5
	Inability of the public sector to develop and manage the PPP process	3.39	6
	Unavailability of large construction companies that can implement PPP projects in some regions	3.37	7
	Lack of suitable skills, experience, and expertise in both the public sector and among private investors	3.36	8
	Difficulty in specifying work requirements and the quality of service	3.34	9
	Provision of incomplete up-front project information by the public sector	3.28	10

The analysis of the rankings based on the barriers’ mean values, as rated by the respondents, showed that they ranged from 2.77 to 3.85 (Table 2). Legal barriers had the highest mean value, with a score of 3.85. This shows that the stakeholders perceived legal barriers to be the biggest hindrance to PPP project implementation in the Saudi healthcare sector, followed by environmental, technological, economic, social and political barriers, respectively.

Delays in approval were considered the biggest legal barrier, with a mean value of 4.06, then law and regulation changes, with a mean value of 3.97. Among environmental barriers, lack of transparency and accountability and lengthy delays in negotiations were considered the leading environmental barriers. The shortage of professionals qualified to handle PPP projects and lack of clarity in the process of implementing PPPs were identified as the two biggest technological barriers, with mean values of 3.78 and 3.69 respectively. Issues with delays in receiving payments and the inability of local institutions to provide long-term financing ranked as the biggest economic barriers, with mean values of 3.64 and 3.53 respectively. Potential conflicts of interests among stakeholders had the highest mean value, 3.81, of the social barriers. Inadequate experience with PPPs and poor understanding of PPPs by decision-makers were the biggest political barriers, with mean values of 3.29 and 3.22, respectively.

Table 4 shows whether various groups of respondents in this study ranked the categories differently and revealed a difference in the perceptions of public-sector officials, consultants and private investors.

**Table 4:** Kruskal-Wallis test of homogenous rankings by groups.

	Government leaders	Public sector officials	Private Investors	Lenders/ Financial	Consultants	Contractors
Number of respondents	10	40	4	6	6	6
Kruskal-Wallis H	9	32	14	4	13	9
Degree of freedom	5	5	5	5	5	5
Asymptotic significance	0.11	<0.001	0.02	0.57	0.03	0.11

## Discussion

This study’s findings showed how the respondents ranked the various barriers and that different stakeholders perceive different barrier categories as having different degrees of impact on the success of PPP projects in the healthcare sector in KSA. The evidence showed that the top three barriers, as rated by the respondents, were legal barriers such as delays in receiving approval and permits and law and regulation changes; environmental barriers such as lack of transparency and accountability; and technological barriers such as a shortage of professionals qualified to handle PPP projects.

Our findings related to the legal barrier agree with those of other studies that have showed that the lack of adequate legal and regulatory frameworks pose a challenge to the successful implementation of PPPs [9,56–58]. A stable regulatory framework that can be easily enforced is essential. KSA’s authorities should study the Middle East and North Africa (MENA) countries’ detailed experiences of good practice in the regulatory frameworks of PPPs. The weakest points of the regulation frameworks in the MENA region are the regulations related to the first step of PPP implementation, the preparation stage. However, there are huge discrepancies between countries. The regulations relating to the first step of implementation of PPPs in Egypt are quite good, whereas, in Iraq and Lebanon they are very poor [57].

Weak/poor regulatory frameworks and enforcement, problems with administrative procedures and guidelines and weak institutional capacity and PPP strategies were identified as strong legal barriers in KSA. Well prepared institutions and a strong legal framework will mitigate delays in approvals and permits, which are the most highly ranked barriers in our study. A well-designed institutional support, the Private Infrastructure Investment Management Center (PIMAC), was introduced in Korea and it would be beneficial to take advantage of its experience. Its functions include (a) provision of advice on structuring PPPs; (b) appraisal of government-sponsored PPP projects and (c) provision of assistance to government agencies in negotiation with private sector PPP developers [59]. It is valuable in preparing any regulatory framework to observe solutions from other countries or agencies and to adopt good practices, while avoiding bad ones [60].

Environmental issues were ranked as the second-highest barriers to the successful implementation of PPPs, with lack of accountability and transparency being the highest-ranked environmental barrier to the success of PPPs in the healthcare sector in KSA, which is not the case in other countries [9]. Concerns are also often raised about delays in negotiations among primary stakeholders. Another barrier is an environment that does not facilitate the implementation of PPPs. This finding echoes those of another study in which it was suggested that it is crucial for governments to create an environment that enables PPPs to become an attractive option [9].

PPPs are like all businesses; they thrive in regions where the laws, regulations and business climate are favourable. In the case of KSA, this study’s results imply that KSA should devise standards that ensure that all parties concerned with the implementation of PPPs are accountable and transparent, both to the public and to each other, so that trust can be established. In addition, laws and policies should be created to facilitate private-sector engagement with the government, with the goal of implementing PPP projects in the healthcare sector.

This study’s results also showed that, in KSA, technological barriers are an important element affecting the success of PPPs in the healthcare sector. It is suggested that a lack of professionals with the right experience and skills and project-preparation capacity are obstacles to the successful implementation of PPPs [61,62]. Similar results were obtained in other PPP studies in Kuwait and Qatar [58]. It is, therefore, recommended that the government should draw on the experience and skills of professionals in developed economies to build and structure its PPPs and, thus, make such projects more attractive to the private sector [63].

In addition, the KSA government should work to improve the capacity of the stakeholders involved in the implementation of PPPs so that the government officials handling such projects can acquire the necessary skills and ability to negotiate proper contracts and become more accountable and transparent. Furthermore, the government can facilitate the process by streamlining such projects into programs, so that they can be prioritised and the private sector will not become overwhelmed by a multiplicity of projects. A good example of a matured user of PPP in healthcare in another country, from which we can learn, is Moheb Hospitals in Iran [64].

The results also showed that there are important political barriers that hinder the successful implementation of PPPs in the healthcare sector. Our study found that the most important barriers are inadequate experience with PPPs and poor understanding of PPPs by decision-makers. Both these barriers in KSA were confirmed by findings from another study that found that the resistance stems from top government officials, who still think that PPP is synonymous to privatization which entails a full divestiture of assets to the private sector [58]. Similar barriers were found to limit the adoption of PPP projects in Singapore [65]. It can be argued that insufficient involvement and the government’s inability to manage PPP projects result in project failures [31]. Furthermore, Gibson and Davies [66] have argued that, in mature economies, local political opposition is an obstacle to implementing PPPs.

In addition, there are economic barriers that make it difficult to implement PPPs. Our findings are similar to those of another study, which suggested that high bidding and transaction costs are also obstacles to the implementation of PPP projects [67,68]. To encourage the private sector to participate and invest in the health sector, the KSA authorities need to provide incentives, conditions and a background that will attract investors [31]. Another problem related to successful PPP implementation that should be considered with caution is the cost of investment and the transparency of healthcare projects [69].

Social barriers were another factor hampering the effective implementation of PPPs. Because PPPs call for close interaction and discussion between the government and the private sector, such barriers are likely to lead the parties to distrust the processes and, therefore, challenge their acceptance by society at large. In the case of multinational engagements, it has been suggested that cultural impediments can pose serious challenges, especially when cultural competence is lacking [2,70,71]. Therefore, before when starting any PPP project, it is imperative to recognise the public’s interests and goals.

Bearing in mind all the potential barriers already mentioned, it should be noted that, for PPPs in healthcare in Islamic countries to succeed, they should be analysed in the context of shari'ah. An example of a pilot PPP in healthcare is the Konya Integrated Health Campus, which was followed by a very similar shari'ah-compliant PPP, the Manisa Hospital in Turkey [72].

Our work analysed the potential barriers proposed by Babatunde et al. [9] and Li [51]. It would be valuable in future research to find any potential barriers that are specific to KSA. Osei-Kyei and Chan [44]analysed 27 papers of critical success factors and found that, of 57 detected factors, 5 were present in many countries, which were appropriate risk allocation and sharing, strong private consortia, political support, community/public support and transparent procurement. Other factors were less common, which means that they are not specific to all countries.

A few limitations should be considered when interpreting this study’s findings. The study used a purposive sample that was relatively small but suitable for the purpose of the study. The study is, therefore, limited by the sample size, and any future study aiming to corroborate these results should employ a larger sample. Moreover, KSA only has limited experience in PPPs in healthcare because projects have just started. Hence, the different stakeholders’ views presented in this study might be limited by their current experiences. With more experience following the implementation of some projects in KSA, in-depth investigations and further research will be warranted.

## Conclusions

The authorities in KSA should benefit from the good practices in regulating the procedures of introducing PPPs, including the preparation of PPPs and procurement and contract management, found in other developing countries [57,72]. It is very important to implement rigorous assessments for the preparation of PPP projects [47]. An economic cost-benefit analysis should be prepared and studies into socioeconomic impact, fiscal affordability, risk identification, financial viability and comparative assessments (PPP versus traditional procurement) and market assessment should be undertaken. These matters should be kept updated while the project is in progress, if necessary. The management processes and practices are crucial to the creation of social value [73].

Should PPPs be more common in healthcare in KSA? If so, then on what scale and which model should be used first? What should be done to ensure that the implementation of projects is successful, thereby contributing to an increase in the level of healthcare services provided? Will their wider application support the efforts made towards Universal Health Coverage? In order to find answers to these questions, one can draw on the experiences of other countries. However, it is necessary to consider each country’s peculiarities and its inhabitants, which might make the copying of solutions effective in certain legal and cultural systems, while, in a different environment, copying might prove useless. The public management models functioning in individual countries are shaped by local, cultural, institutional and legal conditions. An attempt to create a model without considering internal and external conditions is doomed to failure. Whether a PPP can prove to be a useful tool for the socio-economic policy pursued by the Saudi Arabian authorities can only be determined by a careful attempt to put this model into practice, prepared carefully, with an understanding of the essence of the partnership and, above all, according to local conditions.

## List of abbreviations

KSA: Kingdom of Saudi Arabia
MOH: Ministry of Health
PPPs: Public-Private Partnerships

## References

[1] Clifton C, Duffield CF (2006) Improved PFI/PPP service outcomes through the integration of Alliance principles. International Journal of Project Management 24: 573–586.

[2] Klijn E-H, Edelenbos J, Hughes M (2007) Public-private partnership: A two-headed reform. A comparison of PPP in England and the Netherlands New Public Management in Europe: Springer pp. 71–89.

[3] Osborne S (2000) Public-private partnerships: Theory and practice in international perspective: Routledge.

[4] Rosenau PV (2000) Public-private policy partnerships: MIT Press.

[5] Al-Hanawi MK, Qattan AM (2019) An Analysis of Public-Private Partnerships and Sustainable Health Care Provision in the Kingdom of Saudi Arabia. Health services insights 12: 1–10. 10.1177/1178632919859008 PMC6613064 31308685

[6] Chowdhury AN, Chen PH, Tiong RL (2011) Analysing the structure of public–private partnership projects using network theory. Construction management and economics 29: 247–260.

[7] Kosycarz EA, Nowakowska BA, Mikołajczyk MM (2019) Evaluating opportunities for successful public–private partnership in the healthcare sector in Poland. Journal of Public Health 27: 1–9.

[8] Roehrich JK, Lewis MA, George G (2014) Are public–private partnerships a healthy option? A systematic literature review. Social science & medicine 113: 110–119. 24861412 10.1016/j.socscimed.2014.03.037

[9] Babatunde SO, Perera S, Zhou L, Udeaja C (2015) Barriers to public private partnership projects in developing countries: A case of Nigeria. Engineering, Construction and Architectural Management 22: 669–691.

[10] Roehrich J, Barlow JG, Wright S (2013) Delivering European healthcare infrastructure through public-private partnerships: The theory and practice of contracting and bundling In: Das TK, editor. Managing Public-Private Strategic Alliances. 1 ed: Information Age Publishing.

[11] Al-Hanawi MK, Alsharqi O, Almazrou S, Vaidya K (2018) Healthcare finance in the Kingdom of Saudi Arabia: a qualitative study of householders’ attitudes. Applied health economics and health policy 16: 55–64. 10.1007/s40258-017-0353-7 28933057 PMC5797208

[12] Forest JJ, Sousa MV (2006) Oil and terrorism in the New Gulf: framing US energy and security policies for the Gulf of Guinea: Lexington Books.

[13] Walston S, Al-Harbi Y, Al-Omar B (2008) The changing face of healthcare in Saudi Arabia. Annals of Saudi medicine 28: 243–250. 10.5144/0256-4947.2008.243 18596400 PMC6074349

[14] Al-Hanawi MK, Vaidya K, Alsharqi O, Onwujekwe O (2018) Investigating the willingness to pay for a contributory National Health Insurance Scheme in Saudi Arabia: a cross-sectional stated preference approach. Applied health economics and health policy 16: 259–271. 10.1007/s40258-017-0366-2 29307076 PMC5874278

[15] Almalki M, FitzGerald G, Clark M (2011) Health care system in Saudi Arabia: an overview. Eastern Mediterranean Health Journal 17: 784–793. 10.26719/2011.17.10.784 22256414

[16] Mufti MH (2000) Healthcare development strategies in the Kingdom of Saudi Arabia. New York: Springer Science & Business Media.

[17] WHO (2006) Country Cooperation Strategy for WHO and Saudi Arabia 2006–2011. New York: World Health Organization

[18] Elachola H, Memish ZA (2016) Oil prices, climate change—health challenges in Saudi Arabia. The Lancet 387: 827–829. 10.1016/S0140-6736(16)00203-8 PMC7138019 26827075

[19] NTP (2016) Vision 2030: National Transformation Program 2020. Available from: https://vision2030.gov.sa/sites/default/files/NTP_En.pdf [Accessed 18 October 2019].

[20] Barlow J, Roehrich J, Wright S (2013) Europe sees mixed results from public-private partnerships for building and managing health care facilities and services. Health Affairs 32: 146–154. 10.1377/hlthaff.2011.1223 23297282

[21] Yescombe ER (2011) Public-private partnerships: principles of policy and finance: Elsevier.

[22] Commission of the European Communities (2004) Green paper on public-private partnerships and community law on public contracts and concessions. Office for Official Publications of the European Communities. Available from: https://op.europa.eu/en/publication-detail/-/publication/94a3f02f-ab6a-47ed-b6b2-7de60830625e/language-en [Accessed 20 Feb 2020].

[23] Khanom NA (2010) Conceptual issues in defining public private partnerships (PPPs). International Review of Business Research Papers 6: 150–163.

[24] Hodge GA, Greve C (2007) Public–private partnerships: an international performance review. Public administration review 67: 545–558.

[25] Hodge GA, Greve C, Boardman AE (2010) Introduction: the PPP phenomenon and its evaluation In: Hodge GA, Greve C, Boardman AE, editors. International Handbook on Public-Private Partnerships. Cheltenham: Edward Elgar Publishing pp. 3–16.

[26] The World Bank (2015) What are Public Private Partnerships? Washington D.C: The World Bank Group Available from: https://ppp.worldbank.org/public-private-partnership/overview/what-are-public-private-partnerships [Accessed 25 Feb 2020]

[27] Reijneveld SA (2012) Public–private partnerships in public health research: does it work and if yes, how? European Journal of Public Health 22: 165–166. 10.1093/eurpub/ckr187 22158913

[28] Gottwald M, Becker A, Bahr I, Mueller‐Fahrnow A (2016) Public–private partnerships in lead discovery: overview and case studies. Archiv der Pharmazie 349: 692–697. 10.1002/ardp.201600078 27335205

[29] Karawajczyk A, Orrling KM, de Vlieger JS, Rijnders T, Tzalis D (2017) The european lead factory: A blueprint for public–private partnerships in early drug discovery. Frontiers in medicine 3: 75 10.3389/fmed.2016.00075 28154815 PMC5243859

[30] Granados Moreno P, Joly Y, Knoppers BM (2017) Public–private partnerships in cloud-computing services in the context of genomic research. Frontiers in medicine 4: 3 10.3389/fmed.2017.00003 28164085 PMC5247451

[31] Kwak YH, Chih Y, Ibbs CW (2009) Towards a comprehensive understanding of public private partnerships for infrastructure development. California management review 51: 51–78.

[32] Sadeghi A, Barati O, Bastani P, Daneshjafari D, Etemadian M (2016) Strategies to develop and promote public-private partnerships (PPPs) in the provision of hospital services in Iran: a qualitative study. Electronic physician 8: 2208 10.19082/2208 27279993 PMC4886559

[33] PPP Knowledge Lab (2020). Health. Available from: https://pppknowledgelab.org/sectors/health [Accessed 1 March 2020].

[34] Kivleniece I, Quelin BV (2012) Creating and capturing value in public-private ties: A private actor's perspective. Academy of Management Review 37: 272–299.

[35] European Union (2013) Health and economics analysis for an evaluation of the public private partnerships in health care delivery across EU. Annexes. Available from: https://ec.europa.eu/health/expert_panel/sites/expertpanel/files/ppp_finalreport_en.pdf [Accessed 1 March 2020].

[36] Välilä T (2005) How expensive are cost savings? On the economics of public-private partnerships. EIB papers 10: 95–119.

[37] Vecchi V, Hellowell M, Della Croce R, Gatti S (2017) Government policies to enhance access to credit for infrastructure-based PPPs: an approach to classification and appraisal. Public Money & Management 37: 133–140.

[38] Lonsdale C, Watson G (2007) Managing contracts under the UK's Private Finance Initiative: evidence from the National Health Service. Policy & Politics 35: 683–700.

[39] Barlow J, Köberle-Gaiser M (2009) Delivering innovation in hospital construction: Contracts and collaboration in the UK's private finance initiative hospitals program. California management review 51: 126–143.

[40] Vian T, McIntosh N, Grabowski A (2017) “It Keeps Us from Putting Drugs in Pockets”: How a Public-Private Partnership for Hospital Management May Help Curb Corruption. The Permanente Journal 21. 10.7812/TPP/16-113 PMC5528800 28746025

[41] OECD (2015) Consequences of Corruption at the Sector Level and Implications for Economic Growth and Development: OECD Publishing.

[42] McKee M, Edwards N, Atun R (2006) Public-private partnerships for hospitals. Bulletin of the World Health Organization 84: 890–896. 17143463 PMC2627548

[43] Hellowell M (2018) Public Private Partnerships and the Quality and Efficiency of Healthcare Services In: Vecchi V, Hellowell M, editors. Public-Private Partnerships in Health: Springer pp. 1–13.

[44] Osei-Kyei R, Chan AP (2015) Review of studies on the Critical Success Factors for Public–Private Partnership (PPP) projects from 1990 to 2013. International Journal of Project Management 33: 1335–1346.

[45] Chan AP, Lam PT, Chan DW, Cheung E, Ke Y (2009) Potential obstacles to successful implementation of public-private partnerships in Beijing and the Hong Kong special administrative region. Journal of Management in Engineering 26: 30–40.

[46] Yuan J, Zeng AY, Skibniewski MJ, Li Q (2009) Selection of performance objectives and key performance indicators in public–private partnership projects to achieve value for money. Construction management and economics 27: 253–270.

[47] Zheng J, Roehrich JK, Lewis MA (2008) The dynamics of contractual and relational governance: evidence from long-term public–private procurement arrangements. Journal of purchasing and supply management 14: 43–54.

[48] Wright S, Barlow J, Roehrich K (2019) PPPs for Health Services: Public Private Partnerships: Construction, Protection and Rehabilitation of Critical Healthcare Infrastructure in Europe In: Clark R, Hakim S, editors. Public Private Partnerships: Construction, Protection, and Rehabilitation of Critical Infrastructure. New York: Springer.

[49] MOH (2019) Healthcare Transformation Strategy: Private Sector Participation. Available from: https://www.moh.gov.sa/en/Ministry/vro/Private-Sector-Participation/Pages/default.aspx [Accessed 1 December 2019].

[50] MOH (2020) Radiology Pilot Project. Available from: https://www.moh.gov.sa/en/Ministry/vro/Private-Sector-Participation/Work-Streams/Radiology/Pages/structure.aspx [Accessed 25 Feb 2020].

[51] Li B (2003) Risk management of PPP projects. PhD diss Glasgow Caledonian University.

[52] Zikmund W (2003) Business Research Methods. USA: Thomson Learning. South Western.

[53] Garson D (2009) Reliability Analysis: Statnotes from North Carolina State University. Available from: https://faculty.chass.ncsu.edu/garson/PA765/reliab.htm [Accessed 1 October 2019].

[54] Nunally J, Bernstein I (1978) Psychometric theory. New York: McGraw-Hill.

[55] Kumar R (2019) Research methodology: A step-by-step guide for beginners: Sage Publications Limited.

[56] Mouraviev N, Kakabadse NK (2015) Legal and regulatory barriers to effective public-private partnership governance in Kazakhstan. International Journal of Public Sector Management 28: 181–197.

[57] The World Bank (2017) Benchmarking Public-Private Partnerships Procurement: Assessing Government Capability to Prepare, Procure and Manage PPPs. Available from: http://documents.worldbank.org/curated/en/463961476780341706/pdf/109245-WP-PPPBenchmarking-PUBLIC.pdf [Accessed 15 Feb 2020].

[58] Biygautane M (2017) Infrastructure public–private partnerships in Kuwait, Saudi Arabia, and Qatar: Meanings, rationales, projects, and the path forward. Public Works Management & Policy 22: 85–118.

[59] Tsukada S (2019) Vulnerabilities of BOT Scheme and Ways to Overcome Them Under PPP: Cross-Country Comparison of PPP Systems Among Selected Countries. Public Works Management & Policy 24: 284–300.

[60] Boyer EJ, Newcomer KE (2015) Developing government expertise in strategic contracting for public–private partnerships. Journal of Strategic Contracting and Negotiation 1: 129–148.

[61] Mahalingam A (2009) PPP experiences in Indian cities: barriers, enablers, and the way forward. Journal of Construction Engineering and Management 136: 419–429.

[62] Li B, Akintoye A, Edwards PJ, Hardcastle C (2005) Perceptions of positive and negative factors influencing the attractiveness of PPP/PFI procurement for construction projects in the UK: Findings from a questionnaire survey. Engineering, Construction and Architectural Management 12: 125–148.

[63] Holden C (2009) Exporting public–private partnerships in healthcare: export strategy and policy transfer. Policy Studies 30: 313–332.

[64] Miremadi M, Goudarzi K (2019) Developing an innovative business model for hospital services in Iran: a case study of Moheb Hospitals. Leadership in Health Services 32: 129–147. 10.1108/LHS-10-2017-0063 30702041

[65] Hwang B-G, Zhao X, Gay MJS (2013) Public private partnership projects in Singapore: Factors, critical risks and preferred risk allocation from the perspective of contractors. International Journal of Project Management 31: 424–433.

[66] Gibson H, Davies B (2008) The impact of public private partnerships on education: a case study of Sewell Group Plc and Victoria Dock Primary School. International Journal of Educational Management 22: 74–89.

[67] Chan D, Chan A, Lam P. A feasibility study of the implementation of public private partnership (PPP) in Hong Kong; 10–13 April 2006; Hong Kong.

[68] Pollock AM, Dunnigan M, Gaffney D, Macfarlane A, Majeed FA (1997) What happens when the private sector plans hospital services for the NHS: three case studies under the private finance initiative. Bmj 314: 1266 10.1136/bmj.314.7089.1266 9154034 PMC2126590

[69] Acerete B, Stafford A, Stapleton P (2012) New development: New global health care PPP developments—a critique of the success story. Public Money & Management 32: 311–314.

[70] Weththasinghe K, Gajendran T, Brewer G (2016) Barriers in proper implementation of public private partnerships (PPP) in Sri Lanka. 40th AUBEA 2016 Radical Innovation in the Built Environment

[71] Gunnigan L, Rajput R. Comparison of Indian PPP construction industry and European PPP construction industry: process, thresholds and implementation; 2010; Salford: UK.

[72] The World Bank (2017) Mobilizing islamic finance for infrastructure public-private partnerships. Washington D.C: The World Bank Group.

[73] Caldwell ND, Roehrich JK, George G (2017) Social value creation and relational coordination in public‐private collaborations. Journal of Management Studies 54: 906–928.

